# Nonlinear coupling of closely spaced modes in atomically thin MoS_2_ nanoelectromechanical resonators

**DOI:** 10.1038/s41378-024-00844-9

**Published:** 2024-12-27

**Authors:** S M Enamul Hoque Yousuf, Steven W. Shaw, Philip X.-L. Feng

**Affiliations:** 1 Department of Electrical and Computer Engineering, University of Florida, Gainesville, FL 32611 USA; 2 Department of Mechanical and Civil Engineering, Florida Institute of Technology, Melbourne, FL 32901 USA; 3 Department of Mechanical Engineering, Department of Physics and Astronomy, Michigan State University, East Lansing, MI 48423 USA

**Keywords:** NEMS, Nanosensors, Electrical and electronic engineering

## Abstract

Nanoelectromechanical systems (NEMS) incorporating atomic or molecular layer van der Waals materials can support multimode resonances and exotic nonlinear dynamics. Here we investigate nonlinear coupling of closely spaced modes in a bilayer (2L) molybdenum disulfide (MoS_2_) nanoelectromechanical resonator. We model the response from a drumhead resonator using equations of two resonant modes with a dispersive coupling term to describe the vibration induced frequency shifts that result from the induced change in tension. We employ method of averaging to solve the equations of coupled modes and extract an expression for the nonlinear coupling coefficient (*λ*) in closed form. Undriven thermomechanical noise spectral measurements are used to calibrate the vibration amplitude of mode 2 (*a*_2_) in the displacement domain. We drive mode 2 near its natural frequency and measure the shifted resonance frequency of mode 1 (*f*_1s_) resulting from the dispersive coupling. Our model yields *λ* = 0.027 ± 0.005 pm^−2^ · μs^−2^ from thermomechanical noise measurement of mode 1. Our model also captures an anomalous frequency shift of the undriven mode 1 due to nonlinear coupling to the driven mode 2 mediated by large dynamic tension. This study provides a direct means to quantifying *λ* by measuring the thermomechanical noise in NEMS and will be valuable for understanding nonlinear mode coupling in emerging resonant systems.

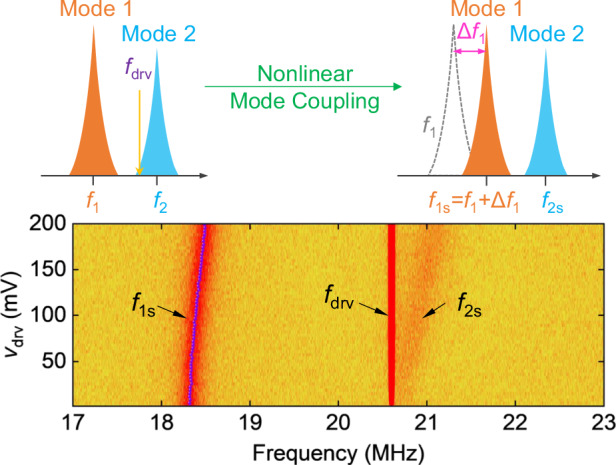

## Introduction

Two-dimensional (2D) materials offer molecularly or atomically thin membranes to realize deeply scaled, exquisite nanoelectromechanical systems (NEMS)^1–3^. Endowed with controllable mechanical degrees of freedom, suspended single- and few-layer 2D materials and their van der Waals (vdW) heterostructures enable NEMS vibrating in their resonant modes that can operate in the elastic membrane limit, where their resonance frequencies are determined by the tension levels in the suspended membranes^3–6^. Such nanoscale resonators often exhibit multimode resonances, and the resonance frequencies exhibit high tunability with external signals, such as electrostatic gate voltage, strain, and Joule heating^4–8^. Thanks to their exceptional strain dependency of resonance frequency, the electrostatic gating induced frequency tunability can be as broad as Δ*f*/*f*_0_ ~ 430%^6^. The resonance frequency is also responsive to external stimuli, including force, mass, pressure, and magnetic field, and thus provides an ideal testbed for studying novel device physics, including nonlinear dynamics, nano-magnetics, and quantum physics^9–14^.

With small driving forces, NEMS resonators operate in the linear regime where the resonance peak (or center) frequency is independent of amplitude, and the signal amplitude versus frequency response curve can be modeled by a square-root-Lorentzian function with a finite quality (*Q*) factor^3,4^. NEMS resonators can be driven into nonlinear regime with moderately strengthened excitation forces, where the resonance peak frequency detunes with the grown amplitude of the response signal at increasing driving force^15–17^. Resonators operating in the nonlinear regime demonstrate many fascinating features including nonlinear mode coupling between different vibrational modes^18^, bistability^19^, parametric resonance^20^, and chaos^21^. Modes with commensurate frequency relationships (*m*:*n*, where *m*,*n* = 1, 2, 3, …) can interact with each other through nonlinear mode coupling which can lead to internal resonances^9,22–25^. With internal resonances, the energy pumped to one mode can be coherently transferred to the other mode or exchanged back and forth between the participating modes. Therefore, the vibration in one mode can significantly affect the other vibrational mode through the phononic energy cycling thanks to nonlinear mode coupling^9,23,26–28^. This energy interchange between involved modes can lead to interesting phenomena, such as phononic frequency combs^9,29^, coherent phonon transfer^9,18,29,30^, improved stability of oscillation^31^, and controlled energy dissipation to the environment^18,30^. In fact, even under relaxed conditions, resonators with closely spaced modes can also demonstrate nonlinear mode coupling and do not require a commensurate frequency relationship between the modes involved^26,32,33^. In previous studies the modes are measured with a phase-locked loop (PLL)^26,32^. To measure the frequency shift of one mode because of intermodal coupling with another mode, the measurement has been typically conducted at low excitation^26^. In this work, by directly measuring the undriven thermomechanical noise spectra of mode 1 arising from its intrinsic Brownian motion, while driving near mode 2, we envision completely avoiding the excitation and driven response of mode 1. This ensures that the frequency shift of mode 1 is not influenced by its own mechanical nonlinearity and the frequency shift is primarily because of the vibration induced tension change by motions of mode 2.

Molybdenum disulfide (MoS_2_), a prominent member of the transition metal dichalcogenide (TMDC) family with high elastic modulus of ~270 GPa (monolayer MoS_2_)^34^, ultralow mass density of 3.3 fg/μm^2^ (monolayer), and reduced phonon-phonon scattering at room temperature^35^ offers an ideal device platform for studying nonlinear dynamics of vibrating 2D mechanical systems. MoS_2_ NEMS resonators are theoretically predicted to have lower dissipation compared to graphene^35^ and demonstrate multimode resonances with reasonably good *Q*s at room temperature^3^. 2D NEMS resonators with closely spaced modes without integer-number commensurate frequency relationships are an intriguing device platform for investigating nonlinear dynamics, as well as to provide a testbed for exploring signal transduction in extremely responsive and miniaturized sensors.

**Fig. 1:**
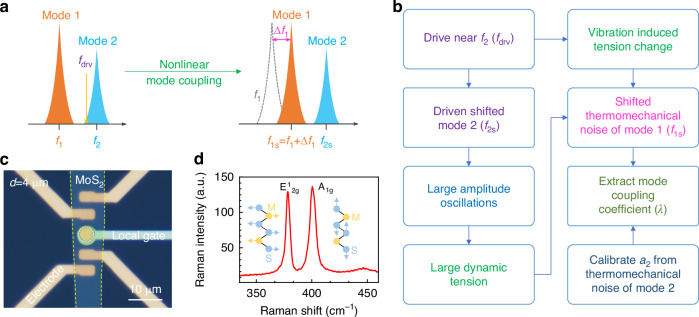
Analysis of nonlinear coupling in closely spaced modes. **a** Illustration of nonlinear mode coupling that induces resonance frequency shift of the undriven mode. *f*_1_ and *f*_2_ are two closely spaced modes and *f*_drv_ is the drive frequency. Δ*f*_1_ represents the shift of mode 1 due to vibration induced tension change in the device. **b** Block diagram delineating the direct approach to determining the mode coupling coefficient *λ* from thermomechanical noise measurement. **c** Optical microscopy image of a bilayer (2L) MoS_2_ NEMS resonator. The yellow dashed line shows the outline of the flake and the white dashed circle in the middle highlights the suspended circular membrane. The lithographically defined electrodes include a set of four-point contacts (golden colored ones) and a buried local gate (platinum colored one) under the circular microtrench. **d** Measured Raman spectra of the suspended 2L MoS_2_ membrane

In this work, we demonstrate nonlinear coupling in closely spaced modes in a bilayer (2L) MoS_2_ NEMS resonator and model the response to understand the dynamics of the resonator. We investigate mode coupling between two modes of the resonator by resolving how the frequency response varies as a function of parameters, such as amplitude of modes, Duffing coefficients, and coupling coefficient, *etc*. The dynamics of the responses are captured by two resonator equations with a single dispersive coupling parameter *λ*. The resonator has mode 1 at *f*_1_ = 18.41 MHz with quality factor *Q*_1_ = 142, and mode 2 at *f*_2_ = 20.45 MHz with *Q*_2_ = 268. We demonstrate the first experiments on measuring *λ* in 2L MoS_2_ 2D NEMS by driving near mode 2 and measuring the undriven thermomechanical noise spectra of mode 1 (Fig. 1a, b). To extract *λ* according to nonlinear mode coupling, we first measure the nonlinearly shifted resonance frequency of mode 1 (*f*_1s_) while driving at fixed (not sweeping) *f*_drv_ near mode 2 but still off resonance of *f*_2_. Under these conditions, from the coupling, *f*_1s_ increases as we increase the driving voltage *v*_drv_ at fixed *f*_drv_. We extract *λ* = 0.027 ± 0.005 pm^−^^2^·μs^−2^, by fitting the calibrated amplitude of mode 2 (*a*_2_) and *f*_1s_. Further, when the fixed *f*_drv_ is on mode 2 resonance while it (*f*_2_) is also being shifted by increased *v*_drv_ at *f*_drv_, we observe and investigate an anomalous resonance frequency shift of mode 1 as it interacts with the driven mode 2 via dynamic tension that is dependent on the resonance motion of mode 2. Specifically, the two-mode interaction mediated by dynamic tension in the device results in a trend in *f*_1s_ vs *a*_2_ that differs from the simple quadratic amplitude dependency (Δ*f*_1_∝ *a*_2_^2^) reported earlier^26,32^, and exhibits a kink in the *f*_1s_ vs *v*_drv_ curve (hence also in the *f*_1s_ vs *a*_2_ curve). Our demonstration offers a direct means of modeling and quantifying nonlinear mode coupling from probing undriven thermomechanical noise spectra in NEMS resonators.

## Analysis of nonlinear coupling in closely spaced modes

To describe nonlinear coupling between two closely spaced modes, an important resonant coupling term comes from the dispersive coupling potential *U*_c_ = ½ *λ x*_1_^2^ *x*_2_^2^, where *x*_1_ and *x*_2_ denote displacements of mode 1 and mode 2, respectively. The fundamental governing equations of motion to represent the coupled resonant modes, including the Duffing nonlinearity and the nonlinear mode coupling, can be expressed as^9^

1 $${\ddot{x}}_{i}+2{\gamma }_{i}{\dot{x}}_{i}+{\omega }_{i}^{2}{x}_{i}+{k}_{3i}{x}_{i}^{3}+\frac{\partial {U}_{c}}{\partial {x}_{i}}={F}_{i},i=1,2$$

Here subscript *i* denotes the mode number, *m*_*i*_, *ω*_*i*_, *k*_3*i*_, and ${\gamma }_{i}={\omega }_{i}/\left(2{Q}_{i}\right)={\omega }_{i}{\zeta }_{i}$ represent effective mass, eigenfrequency, Duffing coefficient, and decay rate ($\zeta$: damping ratio, *Q*: quality factor) respectively, of the *i*^th^ mode. To determine *λ*, we drive one mode and measure the response of the other mode. If we drive near mode 2 and measure mode 1, we can rewrite Eq. (1) for mode 1 and mode 2 as follows:

2 $${\ddot{x}}_{1}+2{\gamma }_{1}{\dot{x}}_{1}+{\omega }_{1}^{2}{x}_{1}+{k}_{31}{x}_{1}^{3}+\lambda {x}_{1}{x}_{2}^{2}=0,$$

3 $${\ddot{x}}_{2}+2{\gamma }_{2}{\dot{x}}_{2}+{\omega }_{2}^{2}{x}_{2}+{k}_{32}{x}_{2}^{3}+\lambda {x}_{2}{x}_{1}^{2}={F}_{2}\cos \left(\omega t\right)$$

Since the driving frequency *ω* is near *ω*_2_, we utilize method of averaging to derive equations that approximate the slow variations in the amplitudes and phases of the *x*_*i*_(*t*) under the conditions of a small and near-resonance drive, low damping, and weak nonlinear effects. To transform Eqs. (2) and (3) into suitable forms for performing the method of averaging, the van der Pol transformation is employed^36^. We express the amplitude and phase equations in a rotating reference frame as follows:

4 $${x}_{1}\left(t\right)={a}_{1}\left(t\right)\cos \left[\omega t+{\phi }_{1}\left(t\right)\right]$$

5 $${x}_{2}\left(t\right)={a}_{2}\left(t\right)\cos \left[\omega t+{\phi }_{2}\left(t\right)\right]$$

6 $${\dot{x}}_{1}\left(t\right)={-\omega a}_{1}\left(t\right)\sin \left[\omega t+{\phi }_{1}\left(t\right)\right]$$

7 $${\dot{x}}_{2}\left(t\right)={-\omega a}_{2}\left(t\right)\sin \left[\omega t+{\phi }_{2}\left(t\right)\right]$$

where *a*_*i*_(*t*) and *ϕ*_*i*_(*t*) represent the time varying amplitude and phase of the displacement signal *x*_*i*_(*t*). The associated constraint equations required to satisfy the transformation in Eqs. (4–7) are

8 $${\dot{a}}_{1}\left(t\right)\cos \left[\omega t+{\phi }_{1}\left(t\right)\right]-{a}_{1}\left(t\right){\dot{\phi }}_{1}\left(t\right)\sin \left[\omega t+{\phi }_{1}\left(t\right)\right]=0$$

9 $${\dot{a}}_{2}\left(t\right)\cos \left[\omega t+{\phi }_{2}\left(t\right)\right]-{a}_{2}\left(t\right){\dot{\phi }}_{2}\left(t\right)\sin \left[\omega t+{\phi }_{2}\left(t\right)\right]=0$$

From Eqs. (6) and (7), we have

10 $${\ddot{x}}_{1}\left(t\right)=-\omega {\dot{a}}_{1}\left(t\right)\sin \left[\omega t+{\phi }_{1}\left(t\right)\right]-\omega {a}_{1}\left(t\right)\left[\omega +{\dot{\phi }}_{1}\left(t\right)\right]\cos \left[\omega t+{\phi }_{1}\left(t\right)\right]$$

11 $${\ddot{x}}_{2}\left(t\right)=-\omega {\dot{a}}_{2}\left(t\right)\sin \left[\omega t+{\phi }_{2}\left(t\right)\right]-\omega {a}_{2}\left(t\right)\left[\omega +{\dot{\phi }}_{2}\left(t\right)\right]\cos \left[\omega t+{\phi }_{2}\left(t\right)\right]$$

We substitute these versions of ${\ddot{x}}_{1}\left(t\right)$ and ${\ddot{x}}_{2}\left(t\right)$ along with Eqs. (4–7) into Eqs. (2) and (3) to express the equations of motion in terms of ${\dot{a}}_{1}\left(t\right)$, ${\dot{\phi }}_{1}\left(t\right)$, ${\dot{a}}_{2}\left(t\right)$, and ${\dot{\phi }}_{2}\left(t\right)$. The resulting equations and the constraint equations Eqs. (8) and (9) are linear in terms of ${\dot{a}}_{1}\left(t\right)$, ${\dot{\phi }}_{1}\left(t\right)$, ${\dot{a}}_{2}\left(t\right)$, and ${\dot{\phi }}_{2}\left(t\right)$ and are readily solvable. The solution for ${\dot{a}}_{1}\left(t\right)$, ${\dot{\phi }}_{1}\left(t\right)$, ${\dot{a}}_{2}\left(t\right)$, and ${\dot{\phi }}_{2}\left(t\right)$ contains both mean and oscillating terms and are slow under the assumptions given above and below. To remove the fast-oscillating terms, we average the equations over one period of the driving force (*τ* = 2π/*ω*) assuming the dynamic variables do not change significantly over one period. The resulting equations show that with damping (${\gamma }_{1} >$0) the first mode settles to zero (practically, to its thermomechanical noise) and so it is assumed that *a*_1_ reduces to 0. We express $\omega ={\omega }_{2}+\Delta \omega$ and ${\omega }_{1}={\omega }_{2}+\Delta {\omega }_{12}$, where Δ*ω* (with |Δ*ω*| $\ll {\omega }_{2}$) is the offset between the drive and *ω*_2_; Δ*ω*_12_ (with |Δ*ω*_12_| $\ll {\omega }_{2}$) is the difference between *ω*_1_ and *ω*_2_. Under these conditions, the averaged equations are simplified to

12 $${\dot{a}}_{1}=0$$

13 $${\dot{\phi }}_{1}=\frac{2{a}_{2}^{2}\lambda +8\left(\Delta {\omega }_{12}-\Delta \omega \right){\omega }_{2}+{a}_{2}^{2}\lambda \cos \left[2\left({\phi }_{1}-{\phi }_{2}\right)\right]}{8{\omega }_{2}}$$

14 $${\dot{a}}_{2}=-\frac{2{a}_{2}{\gamma }_{2}{\omega }_{2}+{F}_{2}\sin \left({\phi }_{2}\right)}{2{\omega }_{2}}$$

15 $${\dot{\phi }}_{2}=\frac{3{a}_{2}^{3}{k}_{32}-8{a}_{2}\Delta \omega {\omega }_{2}-4{F}_{2}\cos \left({\phi }_{2}\right)}{8{a}_{2}{\omega }_{2}}$$

The frequency of mode 1 is given by ${\dot{\phi }}_{1}$. Mode 1, in addition to being undriven externally and only subject to intrinsic thermodynamical fluctuations, also experiences a small component of the harmonic drive at $\omega$ (not expressed in Eq. (2) or Eq. (12)). Since ${\omega }_{1}\,$ < $\omega$, we can take the mode 1 phase relative to the drive to be ${\phi }_{1}=-\pi$. With this, the first mode nonlinear frequency given in Eq. (13) can be expressed as

16 $${\dot{\phi }}_{1}=\Delta {\omega }_{12}-\Delta \omega +\frac{{a}_{2}^{2}\lambda }{8{\omega }_{2}}[2+{\left(\cos {\phi }_{2}\right)}^{2}-{\left(\sin {\phi }_{2}\right)}^{2}]$$

The first two terms can be simply written as $\Delta {\omega }_{12}-\Delta \omega ={\omega }_{1}-\omega$, whereas the final term is the shift from nonlinear coupling. To determine this shift, we consider the response of mode 2. At steady state, ${\dot{a}}_{2}=0$ and ${\dot{\phi }}_{2}=0$. Therefore, from the steady-state conditions on Eqs. (14) and (15), we can solve for $\sin \left({\phi }_{2}\right)$ and $\cos \left({\phi }_{2}\right)$. Applying a trigonometric identity to these leads to

17 $${F}_{2}=\frac{1}{4}{a}_{2}\sqrt{9{a}_{2}^{4}\,{k}_{32}^{2}-48\,{a}_{2}^{2}\,\Delta \omega {k}_{32}{\omega }_{2}+64\,{\left(\Delta \omega \right)}^{2}\,{\omega }_{2}^{2}+64\,{\gamma }_{2}^{2}\,{\omega }_{2}^{2}}$$

Using the expressions obtained for $\sin \left({\phi }_{2}\right)$, $\cos \left({\phi }_{2}\right)$, and that for *F*_2_ in Eq. (16), to focus on the nonlinear shift, ${\dot{\phi }}_{1}$ becomes

18 $${\dot{\phi }}_{1}={\omega }_{1}-\omega +\frac{\lambda \left[27\,{a}_{2}^{6}\,{k}_{32}^{2}-144\,{a}_{2}^{4}\,\Delta \omega {k}_{32}\,{\omega }_{2}+64\,{a}_{2}^{2}\left\{3{\left(\Delta \omega \right)}^{2}+{\gamma }_{2}^{2}\right\}{\omega }_{2}^{2}\right]}{8\,{\omega }_{2}\,\left[9\,{a}_{2}^{4}\,{k}_{32}^{2}-48\,{a}_{2}^{2}\,\Delta \omega {k}_{32}\,{\omega }_{2}+64\,\left\{{\left(\Delta \omega \right)}^{2}+{\gamma }_{2}^{2}\right\}{\omega }_{2}^{2}\right]}$$

The nonlinearly shifted frequency of mode 1 is therefore given by ${\omega }_{1s}={\dot{\phi }}_{1}+\omega$, where

19 $${\omega }_{1s}=2\pi {f}_{1s}={\omega }_{1}+\frac{\lambda \left[27\,{a}_{2}^{6}\,{k}_{32}^{2}-144\,{a}_{2}^{4}\,\Delta \omega {k}_{32}\,{\omega }_{2}+64\,{a}_{2}^{2}\left\{3{\left(\Delta \omega \right)}^{2}+{\gamma }_{2}^{2}\right\}{\omega }_{2}^{2}\right]}{8\,{\omega }_{2}\,\left[9\,{a}_{2}^{4}\,{k}_{32}^{2}-48\,{a}_{2}^{2}\,\Delta \omega {k}_{32}\,{\omega }_{2}+64\,\left\{{\left(\Delta \omega \right)}^{2}+{\gamma }_{2}^{2}\right\}{\omega }_{2}^{2}\right]}$$

When the resonator is driven exactly at the resonance center of mode 2, Δ*ω* = 0, and this result is simplified to

20 $${\omega }_{1s}={\omega }_{1}+\frac{\lambda {a}_{2}^{2}}{8{\omega }_{2}}$$

(ignoring a correction on the order of ${a}_{2}^{6}$). Here, *ω*_1s_ is quadratic function of *a*_2_ and we can compute *λ* by fitting the experimentally measured frequency of mode 1 while driving near or at mode 2. Note that *λ* here is normalized with the effective mass of mode 2. In a more general case, we can use Eq. (19) to fit *f*_1s_ vs *a*_2_ and extract *λ*.

## Resonator fabrication and characterization

We employ all-dry stamp-transfer techniques to fabricate the 2L MoS_2_ drumhead resonator onto a SiO_2_/sapphire substrate with arrays of prefabricated microcavities and electrodes (Fig. 1c)^9^. The microcavity (microtrench) has a diameter (*d*) of 4 μm and depth ~290 nm, which is engineered to obtain excellent displacement-to-optical-reflectance responsivity^3^. Prefabricated electrodes (a set of 4-point contact leads) on top of the substrate make direct contact with the dry-transferred MoS_2_ membrane and are used to ground the flake during the measurement. Local gate at bottom of the microcavity enables electrostatic excitation of the drumhead with low parasitic capacitance. In Raman scattering, MoS_2_ crystal exhibits one in-plane (E^1^_2g_) and one out-of-plane (A_1g_) phonon modes. We utilize a custom-built Raman spectroscopy system with a Horiba *i*HR550 spectrometer equipped with a 2400 grooves/mm diffraction grating, a liquid nitrogen cooled charged coupled device (CCD), and a green semiconductor laser (wavelength ~532 nm) to measure Raman modes in the suspended MoS_2_ membrane^37^. Two distinct Raman modes are detected at E^1^_2g_ = 378.5 cm^−^^1^ and A_1g_ = 400.4 cm^−1^ with a separation of 21.9 cm^−1^ (Fig. 1d). These confirm that the suspended MoS_2_ membrane is bilayer (2L) in nature and has thickness of ~1.3 nm^38^.

**Fig. 2:**
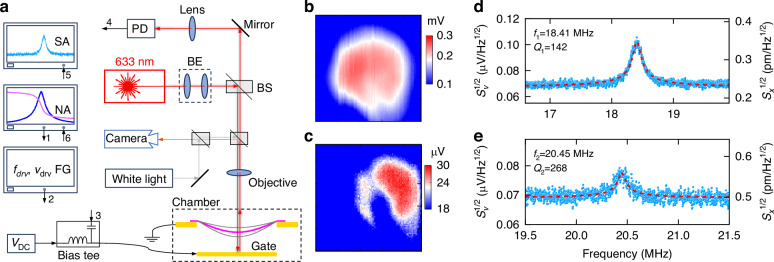
Resonance measurement. **a** Optical interferometry system for measuring the undriven thermomechanical noise spectrum (connect nodes 4, 5 only), driven resonance (connect nodes 1, 3 and 4, 6), and *λ* (connect nodes 2, 3 and 4, 5). PD photodetector, BS beam splitter, BE beam expander, SA spectrum analyzer, NA network analyzer, FG function generator. The dashed-line box at the bottom right illustrates the cross-section of the drumhead resonator. Spatial mapping and visualization of the mode shapes of (**b**) mode 1 and (**c**) mode 2, from location-dependent resonance responses measured while scanning the *x* and *y* directions of the sample stage at *v*_RF_ = 10 mV and *V*_DC_ = 12 V. Measured thermomechanical noise spectra reveal (**d**) mode 1 at *f*_1_ = 18.41 MHz with *Q*_1_ = 142 and (**e**) mode 2 at *f*_2_ = 20.45 MHz with *Q*_2_ = 268. The right axis in (**d**) and (**e**) shows the calibrated signals in displacement domain

To characterize the thermomechanical noise, driven resonance, and nonlinear mode coupling between mode 1 and mode 2 of the 2D NEMS resonator, we employ a customized optical interferometry system as depicted in Fig. 2a. The resonator is driven by a radio frequency (RF) voltage *v*_RF_ from a network analyzer to the local gate of the resonator. The resonance frequency of the NEMS resonator can be tuned by combining a DC gate voltage *V*_DC_ with *v*_RF_ using a bias tee. We employ optical interferometry readout using a 633 nm HeNe laser to detect the resonance motion. A photodetector (PD) transduces the optical signal into electronic signal, which is subsequently fed to the input port of the network analyzer to measure the driven response. We utilize a spectrum analyzer to characterize the undriven (*v*_RF_ signal to the local gate is disconnected) thermomechanical noise spectrum and calibrate the motion of the resonator from voltage domain to the displacement domain. To extract *λ*, we use a function generator to drive near mode 2 resonance while measuring the thermomechanical noise spectra using the spectrum analyzer to determine the blue shifted resonance frequency of mode 1 (*f*_1s_).

To spatially map and visualize the mode shapes of the circular 2D NEMS resonator, we measure the driven responses at different locations on the membrane by scanning the *x* and *y* coordinates of the motorized sample stage^39^. We excite the resonator and collect data by connecting nodes 1, 3 and 4, 6 in the optical interferometry system (Fig. 2a). This way, we create a spatial map of mode 1 (Fig. 2b) and mode 2 (Fig. 2c) of the vibrating drumhead. Due to fabrication imperfections, the spatially mapped modes deviate from the ideal mode shapes of vibrating membrane^39^. The ability to map the mode shapes on a 4 μm-diameter 2D NEMS resonator confirms that our optical interferometry system has high spatial resolution, in addition to detecting the undriven thermomechanical motions with sub-pm/Hz^1/2^ sensitivity.

We then characterize and calibrate the thermomechanical noise spectra of the resonator (connect nodes 4, 5 only). The measured thermomechanical noise can be fitted to a finite-*Q* or damped harmonic resonator model expressed as

21 $${S}_{x}^{\frac{1}{2}}{(\omega)}={\left(\frac{4{k}_{{\rm{B}}}T{\omega }_{n}}{{{m}_{{\rm{eff}}n}}{{Q}_{n}}}\cdot \frac{1}{{\left({\omega }^{2}-{\omega }_{n}^{2}\right)}^{2}+{\left(\frac{\omega {\omega }_{n}}{{Q}_{n}}\right)}^{2}}\right)}^{\frac{1}{2}}$$

Here, *ω*_*n*_ and *m*_eff*n*_ represent the angular resonance frequency and effective mass of mode *n*; *k*_B_ and *T* are Boltzmann’s constant and absolute temperature, respectively. Measurements reveal mode 1 at *f*_1_ = 18.41 MHz with *Q*_1_ = 142 (Fig. 2d) and mode 2 at *f*_2_ = 20.45 MHz with *Q*_2_ = 268 (Fig. 2e), with calibration of the vibrational amplitude from voltage domain to displacement domain. The calculated displacement responsivities of the interferometric optomechanical transduction for mode 1 and mode 2 are *ℜ*_1_ = 295 μV/nm and *ℜ*_2_ = 139 μV/nm, respectively.

## Extraction of nonlinear mode coupling coefficient

**Fig. 3:**
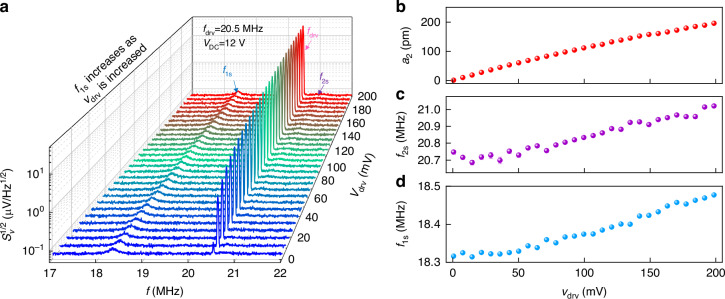
Measured effects of *v*_drv_ at a fixed drive frequency (*f*_drv_ is close to the natural frequency of mode 2) on the driven and undriven thermomechanical noise responses. **a** Measured resonance spectra with varying *v*_drv_ at *f*_drv_ = 20.5 MHz. **b** Evolution of the amplitude of mode 2 versus *v*_drv_. **c** Blue shift in the resonance frequency of mode 2. **d** Blue shift in the resonance frequency of mode 1 due to vibration induced tension change in the device

To probe the undriven thermomechanical noise of mode 1 and achieve *a*_2_ $\gg$ *a*_1_, we employ a function generator to drive near *f*_2_ and measure the resultant spectra using a spectrum analyzer (see Fig. 2a). The output from the function generator (*v*_drv_ at *f*_drv_) is directly connected to the RF input port of the bias tee. This way, we drive near or at mode 2 while measuring the responses of both mode 1 and mode 2. Then we use the displacement responsivity of mode 2 (*ℜ*_2_ = 139 μV/nm) to convert the measured voltage-domain signal to displacement-domain signal and simultaneously record the frequency of mode 1 from its undriven thermomechanical noise resonance. Figure 3a shows the evolution of *f*_1s_, *f*_2s_, and *x*_2_ (in the voltage domain) with increasing *v*_drv_ at *f*_drv_ = 20.5 MHz measured by the spectrum analyzer. We increase the drive strength *v*_drv_ from 1 to 201 mV. At this *f*_drv_, the amplitude of mode 2 (*a*_2_) increases as we increase *v*_drv_ (Fig. 3b). Fitting the data to Eq. (21) provides the shifted resonance frequency of mode 1 (*f*_1s_) and mode 2 (*f*_2s_), which show a blue shift with higher driving voltage (*v*_drv_) (Fig. 3c, d). Analyzing the off-resonance data shows ~1 nV/Hz^1/2^ reduction in the baseline noise level of the system in the blue-shifted mode at *v*_drv_ = 200 mV. *Q* factors fluctuate with a mean 160 and standard deviation 14.

**Fig. 4:**
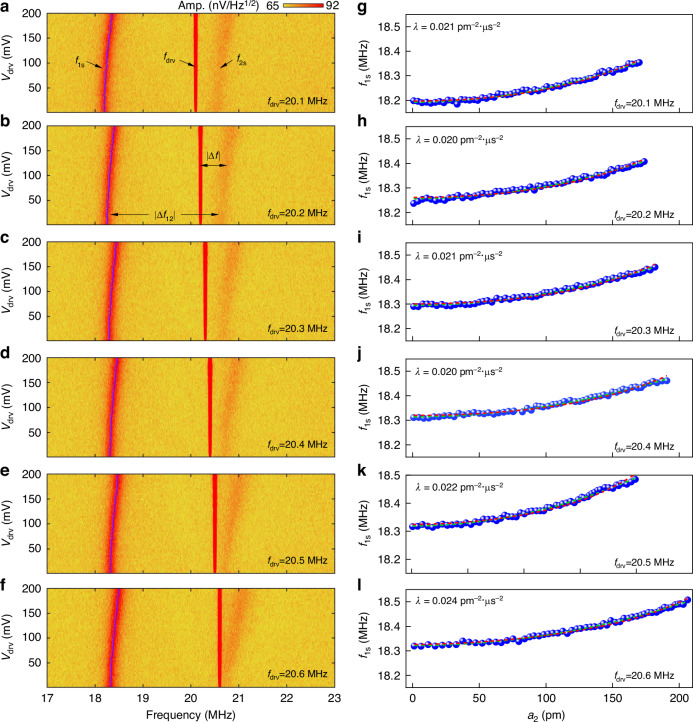
Extraction of mode coupling coefficient. **a**–**f** Color plots showing the evolution of mode 1 with increasing drive voltage *v*_drv_ at varying *f*_drv_. *v*_drv_ (from function generator) is applied to the RF port of the bias tee and the responses are measured using the spectrum analyzer. To show *f*_1s_ or *f*_2s_ with visible thermomechanical noise spectra amplitudes, the higher limit of the color plot is set at 92 nV/Hz^1/2^. Violet dots in each panel represent *f*_1s_ at the given *v*_drv_. **g**–**l** Measured resonance frequency of mode 1 (*f*_1s_) as a function of amplitude of mode 2 (*a*_2_) at varying *f*_drv_. The number in the lower right corner of each panel indicates the respective drive frequency (*f*_drv_) from the function generator. Red and green dashed curves are fitting results to Eqs. (19) and (20), respectively. Green dashed curves are mostly covered by red dashed lines and are barely visible

**Fig. 5:**
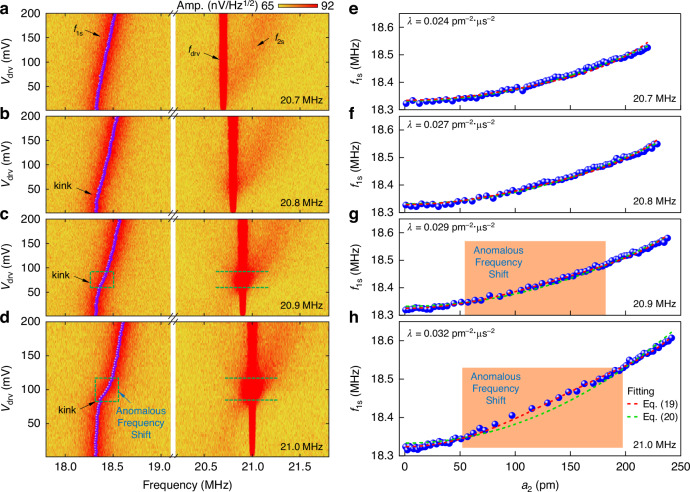
Extraction of mode coupling coefficient and anomalous frequency shift. **a**–**d** Color plots showing the evolution of mode 1 with increasing drive voltage *v*_drv_ at varying *f*_drv_. To show *f*_1s_ or *f*_2s_ with visible thermomechanical noise spectra amplitudes, the higher limit of the color plot is set to be 92 nV/Hz^1/2^. Violet dots in each panel represent *f*_1s_ at the given *v*_drv_. The anomalous frequency shift with a kink is clearly visible in (**d**). **e**–**h** Measured resonance frequency of mode 1 (*f*_1s_) as a function of amplitude of mode 2 (*a*_2_) at varying *f*_drv_. The number in the lower right corner of each panel indicates the respective drive frequency (*f*_drv_) from the function generator. Red and green dashed curves are fitted to Eqs. (19) and (20), respectively. The green dashed lines are almost covered by the red dashed lines in (**e**) and (**f**) as both Eqs. (19) and (20) qualitatively describe the trend when there is no (or visible) anomalous frequency shift in *f*_1_. The shaded regions in (**g**) and (**h**) correspond to the green dashed rectangle in (**c**) and (**d**), respectively, highlighting the anomalous shift in *f*_1s_ when driven mode 2 interacts with mode 1

To extract the nonlinear mode coupling coefficient and investigate the effects of varying *f*_drv_ on *λ*, we conduct a series of experiments as delineated in Figs. 4 and 5. Figures 4a–f show the evolution of *f*_1s_ when we change *v*_drv_ with *f*_drv_ varying from 20.1 to 20.6 MHz. All such experiments exhibit consistent trends when we increase *v*_drv_. The data in Fig. 4a–f are used to derive the data in Fig. 4g–I, where the former figures show *v*_drv_ versus *f* and the latter figures represent *f*_1s_ versus *a*_2_. The resonance widths of mode 1 and mode 2 are *f*_1_/*Q*_1_ = 0.13 MHz and *f*_2_/*Q*_2_ = 0.08 MHz, respectively, and the frequency difference between mode 1 and mode 2 is |Δ*f*_12_| ≈2 MHz. Therefore, the separation between mode 1 and mode 2 is ~25 times the resonance width of mode 2. In our experiments, we are driving ~20 resonance widths away from mode 1 and thus the contribution of the driving force to mode 1 is negligible. As a result, we observe a thermomechanical noise peak for undriven mode 1. When the drive frequency *f* *=* *f*_drv_ is close to the eigen frequency of mode 2, the tension in the membrane causes blue shift of mode 1. As shown in Fig. 4g–I, the red dashed lines fitted to Eq. (19) quantitively describe this trend. In this range of *f*_drv_, we note that these data are well approximated by Eq. (20) as reported in literature (Δ*f*_1_∝ *a*_2_^2^)^26,32^. However, because Δ*f* ≠ 0, we use Eq. (19) to extract the mode coupling coefficient for *f*_drv_ from 20.1 to 20.6 MHz (Fig. 4g–I). As an example, Fig. 4a shows the evolution of *f*_1s_ and *f*_2s_ as we increase the drive voltage at a fixed *f*_drv_ = 20.1 MHz. To extract *λ*, we plot *f*_1s_ versus *a*_2_ as shown in Fig. 4g. *f*_1s_ increases from 18.19 to 18.35 MHz and *a*_2_ increases from 1 to 170 pm as we increase *v*_drv_ from 1 to 201 mV. By fitting the data to Eq. (19), we determine the value of nonlinear coupling coefficient, *λ* = 0.021 pm^−2^·μs^−2^ at *f*_drv_ = 20.1 MHz. Since the fitting curve matches the experimental data very well, the dynamics of the nonlinear system can be accurately captured with the model as described by Eq. (19). The value of *λ* varies between 0.021 pm^−2^·μs^−2^ and 0.024 pm^−^^2^·μs^−^^2^ for *f*_drv_ from 20.1 to 20.6 MHz. Table 1 summarizes the value of *λ* extracted by fitting the data to both Eqs. (19) and (20). The shift of mode 2 is from its Duffing effect and can be calculated with the similar analysis of Eq. (15).

**Table 1:** Summary of extracted *λ* from fitting experimental data to Eqs. (19) and (20)

*f*_drv_ (MHz)	Δ*f* (MHz)	*λ* [Fitting Eq. (19)] (pm^−2^·μs−^2^)	*λ* [Fitting Eq. (20)] (pm^−^^2^·μs^−^^2^)
20.1	−0.35	0.021	0.037
20.2	−0.25	0.020	0.032
20.3	−0.15	0.021	0.031
20.4	−0.05	0.020	0.028
20.5	0.05	0.022	0.028
20.6	0.15	0.024	0.027
20.7	0.25	0.024	0.027
20.8	0.35	0.027	0.027
20.9	0.45	0.029	0.029
21.0	0.55	0.032	0.032

When *f*_drv_ is above 20.6 MHz, mode 1 interacts with the driven mode 2 (Fig. 5). In such a case, Δ*f*_1_ deviates from its simple quadratic form compared to *f*_drv_ ≤ 20.6 MHz as shown in Fig. 4 as well as earlier studies^26,32^. Inside the resonance bandwidth of mode 2, the input RF drive excites mode 2. Large amplitude oscillations of the driven mode 2 lead to large dynamic tension in the membrane. This results in a kink in the observed *f*_1s_ vs *v*_drv_ curve, which we refer to as anomalous frequency shift in *f*_1_. Therefore, a resonance-based tension clearly deviates *f*_1s_ from the response shown in Fig. 4. In such a case, Eq. (20) can no longer fit the data (see the green dashed line in Fig. 5h). But the kink in *f*_1s_ vs *a*_2_ is well captured by our general model in Eq. (19), which can quantitatively describe the anomalous frequency shift. At *f*_drv_ = 21.0 MHz, the response shows such a behavior where the driven mode 2 interacts with the undriven mode 1 from *v*_drv_ ≈ 80 mV to 120 mV, showing a higher frequency shift in *f*_1_ (Fig. 5d). Therefore, we employ generalized Eq. (19) to fit the experimental data and extract *λ* from the fitting. After calibrating *a*_2_ and determining *f*_1s_ for each drive level, we fit the data to Eq. (19) and obtain *λ* = 0.032 pm^−2^·μs^−2^ with *k*_32_ = 0.027 pm^−^^2^·μs^−2^ and *γ*_2_ = 4.9 × 10^6^ s^−1^ (Fig. 5h). The change in the coupling coefficient between two modes at different *f*_drv_ might be attributed to the environmental fluctuations as these measurements have been performed at room temperature in moderate vacuum (~30 mTorr), and the drive voltage sweeping at each drive frequency can take ~30 min to complete. Thermally enhanced softening nonlinearity has been employed to demonstrate tunability of mode coupling in a doubly-clamped MEMS beam resonator^40^. However, with the current interpretation of the model, we do not know the physical origin for the derived mode coupling coefficient variation at different driving conditions.

**Fig. 6:**
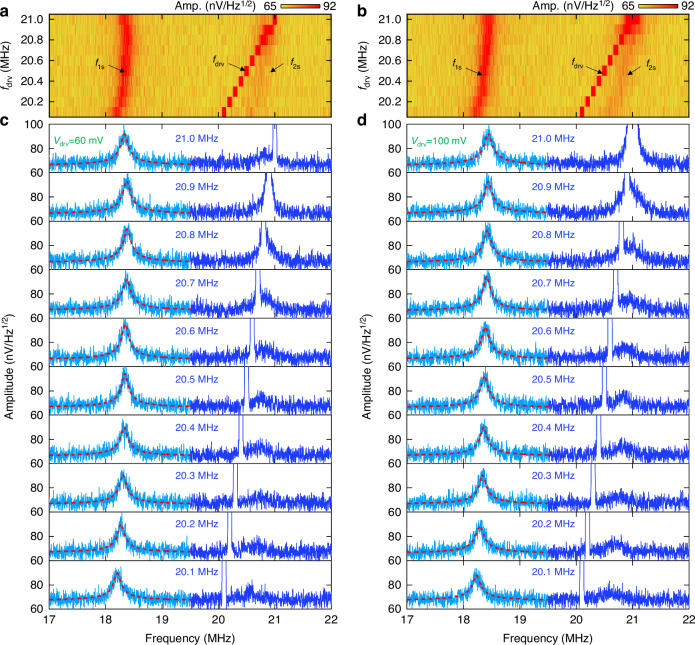
Effect of interaction between *f*_drv_ and *f*_2s_. Measured thermomechanical noise of mode 1 while driving near or at mode 2 at varying *f*_drv_ with (**a**) *v*_drv_ = 60 mV and (**b**) *v*_drv_ = 100 mV. *f*_drv_ and *f*_2s_ cross as we increase *f*_drv_. The interaction due to mode coupling decreases as *f*_drv_ moves away from the eigen frequency of mode 2, resulting in reduced frequency shift in *f*_1_. **c**, **d** show the individual measured spectra used to construct the color maps plots in (**a**, **b**), respectively. The numerical label in each subpanel indicates *f*_drv_. Red dashed lines are fitting curves to Eq. (21)

Figures 6a, b show the effects of different drive frequencies at *v*_drv_ = 60 mV and *v*_drv_ = 100 mV, respectively. Individual spectra used to construct these color plots are shown in Fig. 6c, d. At *v*_drv_ = 60 mV, as we increase *f*_drv_ from 20.1 to 21.0 MHz, *f*_1s_ gradually increases from 18.21 to 18.38 MHz and then drops to 18.33 MHz. The drop in *f*_1s_ can be attributed to lower dynamic tension when mode 2 is not driven. As we repeat the experiment with *v*_drv_ = 100 mV, *f*_1s_ keeps increasing as we vary *f*_drv_ from 20.1 to 21.0 MHz, although the frequency shift slows down. With higher *v*_drv_ > 120 mV, we expect *f*_1s_ to monotonically keep increasing within the 20.1 to 21.0 MHz as mode 2 is undriven. Therefore, when mode 2 is driven, the response deviates from Eq. (20) and we must consider Duffing nonlinearity and linear damping term to describe the nonlinear mode coupling, as expressed in Eq. (19). The interaction of the thermomechanical noise spectra with dispersive coupling and the corresponding linewidth change, as depicted in Figs. 5 and 6, are intriguing. This subject has been analyzed^41^, but its full consideration for the present devices should warrant further study in future work. Note that, in this work, we have not observed any internal resonance. However, if the system is driven hard enough, it will induce modal interaction through 1:1 internal resonance which can lead to coherent energy transfer between the involved modes, and thus may generate phononic frequency combs.

## Conclusions

In summary, we have demonstrated nonlinear coupling in closely spaced modes in a bilayer MoS_2_ 2D NEMS resonator. We have modeled the responses of the resonator using a single dispersive coupling term to describe the vibration induced tension change in the device with the two coupled modes. We have extracted the dispersive mode coupling coefficient *λ* by driving the resonator near mode 2. Our method provides a direct approach to determining the nonlinear mode coupling coefficient from the undriven thermomechanical noise spectra in NEMS resonators and captures the anomalous frequency shift in mode 1. While in the experimental demonstration herein we have employed a bilayer MoS_2_ drumhead resonator, our method and analysis are general and are thus applicable to other materials and MEMS/NEMS resonators as well. Nonlinear mode coupling enables coherent manipulation of energy between modes and can benefit emerging devices such as multimode resonators and phononic frequency combs, and their applications in coherent signal processing and sensing in micro/nanoscale integrated systems.
